# Incremental value of the perivascular fat attenuation index surrounding the superior mesenteric artery on non-contrast CT for predicting SMA abnormalities in patients with acute abdominal pain

**DOI:** 10.3389/fmed.2026.1899182

**Published:** 2026-09-11

**Authors:** Yanan Zhang, Yue Zhou, Jianbing Yin, Jia Liu, Jialei Ming, Lei Cui

**Affiliations:** 1 Department of Radiology, Nantong First People's Hospital, Nantong, Jiangsu, China; 2 Radiology Department, Affiliated Nantong Clinical College of Nantong University, Nantong First People's Hospital, School of Medicine, Nantong University, Nantong, Jiangsu, China

**Keywords:** fat attenuation index, non-contrast CT, perivascular adipose tissue, SMA abnormalities, superior mesenteric artery

## Abstract

**Objective:**

To evaluate the predictive value of the perivascular fat attenuation index (FAI) surrounding the superior mesenteric artery (SMA) on non-contrast CT for SMA abnormalities in acute abdominal pain and FAI's incremental utility in risk models.

**Methods:**

This retrospective study included 262 acute abdominal pain patients (77 with SMA abnormalities, 185 controls) admitted between January 2020 and December 2025. All underwent non-contrast and contrast-enhanced CT within 48 h. Independent predictors were identified via univariate and multivariate logistic regression. The maximum area under the curve (AUC) was obtained through receiver operating characteristic analysis, and the predictive performance of models was comprehensively evaluated using net reclassification (NRI) and integrated discrimination improvement (IDI). Decision curve analyses (DCA) and calibration curves were used to evaluate the clinical utility and calibration of the models.

**Results:**

Independent predictors of SMA abnormalities were smoking (OR = 10.452, 95% CI: 2.528–43.219), elevated high-sensitivity C-reactive protein [hsCRP] (OR = 1.012, 95% CI: 1.001–1.023), increased SMA diameter [SMA-d] (OR = 4.414, 95% CI: 2.758–7.062), and high FAI (OR = 1.092, 95% CI: 1.053–1.132; all *P* < 0.05). Four models were constructed: Model A (smoking + hsCRP), Model B (Model A + SMA-d), Model C (Model A + FAI), and Model D (Model A + SMA-d + FAI). Model D achieved the highest AUC (0.970; 95% CI: 0.952–0.988), outperforming Model A (AUC = 0.734), Model B (AUC = 0.945), and Model C (AUC = 0.878; all *P* < 0.001). Versus Model A, Model D showed superior NRI (0.780) and IDI (0.573; both *P* < 0.001). Model B outperformed Model C in incremental value (NRI: 0.657 vs. 0.457; IDI: 0.460 vs. 0.295; all *P* < 0.001) when each was compared with Model A. DCA confirmed Model D provided maximal net clinical benefit (threshold probability: 0%–91%). The Model D nomogram demonstrated excellent calibration.

**Conclusion:**

A high FAI was independently associated with SMA abnormalities, although its incremental predictive value was lower than that of SMA-d. Thus, FAI might serve as a complementary imaging biomarker rather than a stand-alone diagnostic marker. Integrating smoking status, hsCRP, SMA-d, and FAI might improve early risk stratification based on non-contrast CT and support timely diagnostic evaluation.

## 1 Introduction

Acute abdominal pain is one of the most frequent reasons for patients to seek treatment in the emergency department (1). The superior mesenteric artery (SMA) is a critical vessel in this abdominal pain, as it plays an essential role in supplying blood to the midgut. Acute SMA abnormalities, including SMA dissection (SMAD), thromboembolism (SMAT), aneurysms, pseudoaneurysms, and vasculitis (SMAV), can lead to severe outcomes such as intestinal ischemic necrosis and other potentially life-threatening complications (2). However, the diagnosis of SMA abnormalities remains challenging. Their atypical clinical presentations, combined with the lack of specific biomarkers, increase the risk of misdiagnosis or delayed diagnosis, and patients are frequently treated for other acute abdominal conditions such as acute pancreatitis, acute appendicitis, or gastrointestinal perforation. Misdiagnosis may delay appropriate intervention and ultimately compromise patient outcomes. Therefore, early recognition and prompt management of acute SMA vascular diseases are essential to improve patient prognosis.

Currently, computed tomography angiography (CTA) and contrast-enhanced computed tomography (CECT) are the gold standards for diagnosing abnormalities in the SMA (3–5). Despite their diagnostic value, clinical applications of these imaging modalities are often limited. Several factors contribute to these limitations, including nonspecific clinical manifestations, allergic reactions to contrast agents, the risk of contrast-induced nephrotoxicity, and limited access to 24 h emergency CECT/CTA services, particularly in resource-limited settings. Consequently, non-contrast abdominal computed tomography (CT) is frequently used as the initial imaging modality for patients presenting with acute abdominal pain in both outpatient and emergency settings. Therefore, identifying early imaging indicators of acute mesenteric artery lesions on non-contrast CT is of substantial clinical importance, as this approach may facilitate the timely diagnosis and management of these conditions, which can pose serious threats to patient health.

Perivascular adipose tissue (PVAT) and blood vessels are interconnected through complex vasocrine and paracrine signaling pathways (6), which play important roles in the development, progression, diagnosis, and treatment of cardiovascular and cerebrovascular diseases. PVAT is the adipose tissue surrounding the arteries, and its CT attenuation reflects the inflammatory status of the vascular wall. When arterial inflammation occurs, inflammatory cytokines diffuse into the PVAT, leading to increased CT attenuation (i.e., an elevated fat attenuation index [FAI]) (7, 8). As a noninvasive inflammatory marker derived from non-contrast CT, the FAI has attracted increasing attention in recent years. Previous studies confirmed the association between the FAI and arterial inflammation, and the relationship between PVAT and diseases of the aorta, coronary arteries, and carotid arteries has become a research focus (8–13). According to Tan et al. (14), there is a significant correlation between spontaneous isolated SMAD (SISMAD) and perivascular fat stranding (PFS) and factors such as emergency admissions, abdominal pain occurrence, diagnostic modality, and anatomical subtype. However, the predictive value of PVAT for SMA abnormalities remains unclear.

This study aimed to investigate the utility of the FAI for the rapid identification of SMA abnormalities in patients with acute abdominal pain using non-contrast abdominal CT, evaluate its incremental value in risk prediction, and provide evidence for optimizing the CT diagnostic workflow for acute abdominal pain and the early detection of SMA abnormalities.

## 2 Materials and methods

### 2.1 Study population

Patients experiencing acute abdominal pain (characterized as non-traumatic abdominal pain lasting no longer than five days) who were admitted to Nantong First People's Hospital between January 2020 and December 2025 were retrospectively and consecutively enrolled. The inclusion criteria were: ① non-contrast abdominal CT covering the SMA and subsequent abdominal CECT or SMA CTA performed within 48 h and ② all participants were at least 18 years of age. The exclusion criteria were: ① history of SMA surgery; ② the presence of pancreatitis, peritonitis, abdominal tumors, abdominal aortic dissection, or other conditions involving the main trunk of the SMA, while SMA abnormalities were present only in the branches; ③ incomplete clinical data; or ④ poor CT image quality.

The present study received formal approval from the Ethics Committee of Nantong First People's Hospital (Approval No. 2025-KT387-02), and the requirement for obtaining written informed consent from participants was waived.

### 2.2 Non-contrast CT acquisition

Non-contrast CT images were obtained using two CT systems (Siemens SOMATOM Definition Flash and Siemens SOMATOM Force; Siemens Healthineers, Forchheim, Germany). The scanning parameters were:

- For the Siemens SOMATOM Definition Flash system, the reference tube voltage was configured using CARE Dose4D and CARE kV with automatic tube current modulation. The slice thickness was 1–2 mm, collimation was 1 × 128 × 0.6 mm, rotation time was 0.5 s, and pitch was 0.6.
- For the Siemens SOMATOM Force system, the reference tube voltage was configured similarly using CARE Dose4D and CARE kV, with automatic tube current modulation. The slice thickness was 1–2 mm, collimation was 1 × 192 × 0.6 mm, rotation time was 0.5 s, and pitch was 1.2.

### 2.3 Diagnosis of SMA abnormalities

Table 1 summarizes different types of SMA abnormalities. The definitive diagnostic standard was based on the abdominal CECT or SMA CTA findings. SMA abnormalities were diagnosed through the application of various postprocessing techniques. These methods included curved planar reconstruction, which enhances the visualization of anatomical structures along specific curves; multiplanar reconstruction, which allows viewing of images in multiple planes; maximum intensity projection, which highlights the areas of highest intensity within the dataset; and volume rendering techniques, which provide a comprehensive view of three-dimensional datasets.

**Table 1:** Definitions of various SMA abnormalities.

SMA abnormalities	Definition
SISMAD	(a) Formation of an intimal flap within the SMA; (b) A structure with two lumens is observed in the SMA, where contrast agent fills the false lumen; (c) The SMA may present with ulcer-like protrusions that connect to the lumen or may not; (d) Crescent-shaped intramural hemorrhage located in the SMA (15).
	Dissecting aneurysm: The diameter of the SMAD is 1.5 times larger than the normal size of SMA (16).
SMA aneurysms	(a) Dilation of the SMA, which could either be fusiform or saccular, may exhibit a diameter that is 1.5 times greater than the typical measurement, and could potentially feature ongoing calcification of the wall; (b)Thrombus might also exist within the aneurysm sac (17).
SMA pseudoaneurysms	Localized rupture of the SMA with surrounding encapsulated hematoma, intraluminal thrombus, and discontinuous calcification of the vessel wall (18).
SMAT	Low-density filling defect areas or vascular cutoff sign within the SMA (19).
SMAV	Beaded appearance of the SMA, circumferential thickening of the arterial wall, luminal stenosis, and microaneurysm formation (20).

### 2.4 Clinical data collection

Demographic characteristics and laboratory data were collected for analysis. The data included sex, age, and body mass index. Medical history was recorded with emphasis on diabetes, hypertension, malignancies, and smoking history. To further assess clinical status, laboratory markers were evaluated, including white blood cell (WBC) count, D-dimer, and high-sensitivity C-reactive protein (hsCRP). The analysis also included lipid profile parameters, namely total cholesterol, triglycerides, high-density lipoprotein, low-density lipoprotein, apolipoprotein A-I, apolipoprotein B, and lipoprotein(a).

### 2.5 CT image analysis

**Figure 1:**
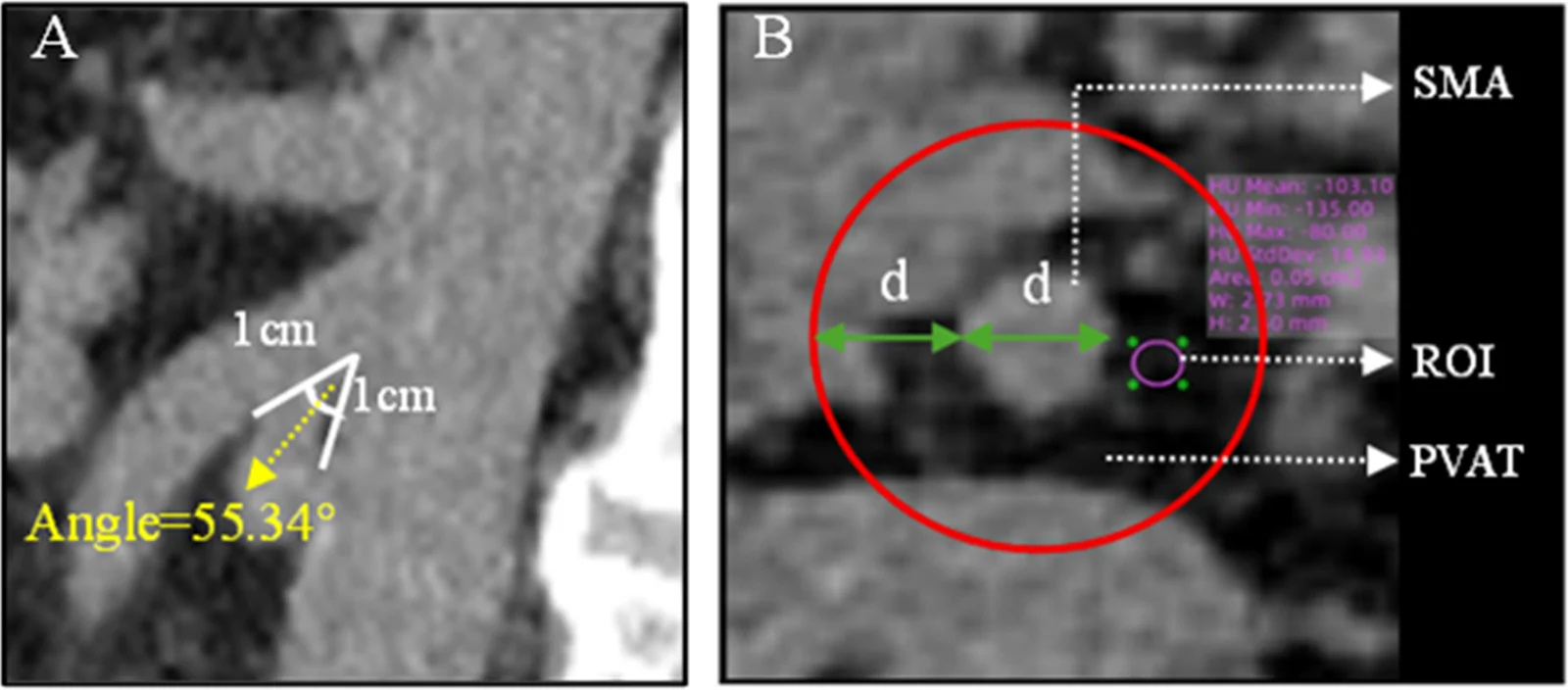
**(A)** The angle formed between the aorta and the superior mesenteric artery (SMA) is defined by the anterior wall of the distal abdominal aorta and the posterior wall of the SMA. **(B)** Schematic illustration of perivascular adipose tissue (PVAT) surrounding the SMA on non-contrast abdominal computed tomography images. PVAT refers to adipose tissue located within a radial distance equal to the vessel diameter (d), with computed tomography attenuation values ranging from −190 HU to −30 HU. ROI, region of interest.

Non-contrast CT images were independently assessed by two radiologists. The first radiologist had three years of experience in the field, while the second radiologist had eight years of expertise specifically in cardiovascular imaging, both of whom were blinded to the final diagnosis. The following parameters were measured: ① SMA diameter (SMA-d); ② mean CT attenuation of the SMA lumen (SMA-mean); ③ aortomesenteric angle (AM-a); ④ presence of SMA wall calcification; ⑤ diameter of abdominal aortic diameter at the level of the SMA origin. The SMA-d was defined as the maximum diameter of the SMA trunk. SMA-mean was defined as the average CT attenuation of the SMA lumen at the maximum diameter. AM-a was measured on sagittal reconstructed images as the angle between the two lines. The first line connected the origin of the SMA to a point 1 cm distal to its posterior wall. The second line connected the same SMA origin to a point located 1 cm distal to the anterior wall of the abdominal aorta, as shown in Figure 1A (21). To ensure measurement reliability, each parameter was measured thrice, and the average of these measurements was calculated and used for subsequent analyses.

### 2.6 Perivascular fat attenuation analysis

To ensure measurement consistency, the FAI was assessed using a standardized window width of 300 Hounsfield units (HU) and window level of 40 HU on non-contrast CT images. The PVAT was characterized as adipose tissue situated within a radial distance corresponding to the vessel diameter being examined. This tissue was identified based on CT attenuation values that fell within the range of −190 HU to −30 HU (8). Beginning at the origin of the SMA, a region of interest (ROI) measuring 5 mm^2^ was placed every 2 cm along the visible PVAT of the SMA trunk, with each ROI positioned at least 1 mm from the outer wall of the SMA (Figure 1B). Both radiologists performed measurements on the same side of the SMA. The FAI value was calculated as the average CT attenuation for all the ROIs. The primary trunk of the SMA was defined as the area extending from its origin to the branching point of the ileocolic artery, the terminal branch of the SMA. The final values were calculated as the mean of the results obtained by the two radiologists. When perivascular inflammation increased fat attenuation and obscured the fat-wall interface, the following procedure was used: (1) the expected anatomical course of the SMA served as a reference; (2) equivocal regions were reviewed by consensus; and (3) a third senior radiologist adjudicated any remaining uncertainty.

### 2.7 Statistical analysis

Statistical analysis was performed using SPSS (version 22.0) and R (version 4.3.1) software. For the continuous variables that exhibited a normal distribution, the data are presented as mean ± standard deviation $\left(\bar{x}\pm s\right)$. Independent-sample *t* tests were used to determine whether there were significant differences between groups. Continuous variables that did not follow a normal distribution are summarized as median and interquartile range and presented in the format M (P25, P75). The Mann–Whitney U test was used to compare non-normally distributed variables across different groups. Categorical variables are expressed as frequencies and percentages. To test for significant differences among these categories, the chi-square (*χ*^2^) test was applied. The interobserver reliability of the CT parameters was assessed using the kappa statistic for categorical variables and the intraclass correlation coefficient (ICC) for continuous variables.

Initially, a univariate analysis was performed to identify potential predictors. Multicollinearity among variables was assessed by calculating the variance inflation factor (VIF) and tolerance. Variables that demonstrated a *VIF* of less than 10 and a tolerance greater than 0.1 were deemed to exhibit acceptable levels of multicollinearity. Therefore, these variables were included in the multivariate logistic regression model for further analyses. For multivariable analysis, a binary logistic regression model was fitted using backward stepwise selection based on the likelihood-ratio test (backward LR), with variables sequentially removed at *P* > 0.05. Receiver operating characteristic (ROC) curve analysis was used to evaluate the diagnostic performance of each model, and the optimal cutoff value corresponding to the maximum area under the curve (AUC) was determined. At this cutoff value, the sensitivity, specificity, positive predictive value, and negative predictive value were calculated. The DeLong test was used to compare the AUCs across different models. The net reclassification index (NRI) and integrated discrimination improvement (IDI) were calculated to assess the reclassification performance of the updated models (22). Decision curve analysis (DCA) was used to assess the net clinical benefits of the models. Finally, model calibration was assessed using the Hosmer-Lemeshow goodness-of-fit test and calibration curves generated from 1,000 bootstrap resamples. A *P* value of less than 0.05 was considered statistically significant.

## 3 Results

### 3.1 Patient characteristics

**Figure 2:**
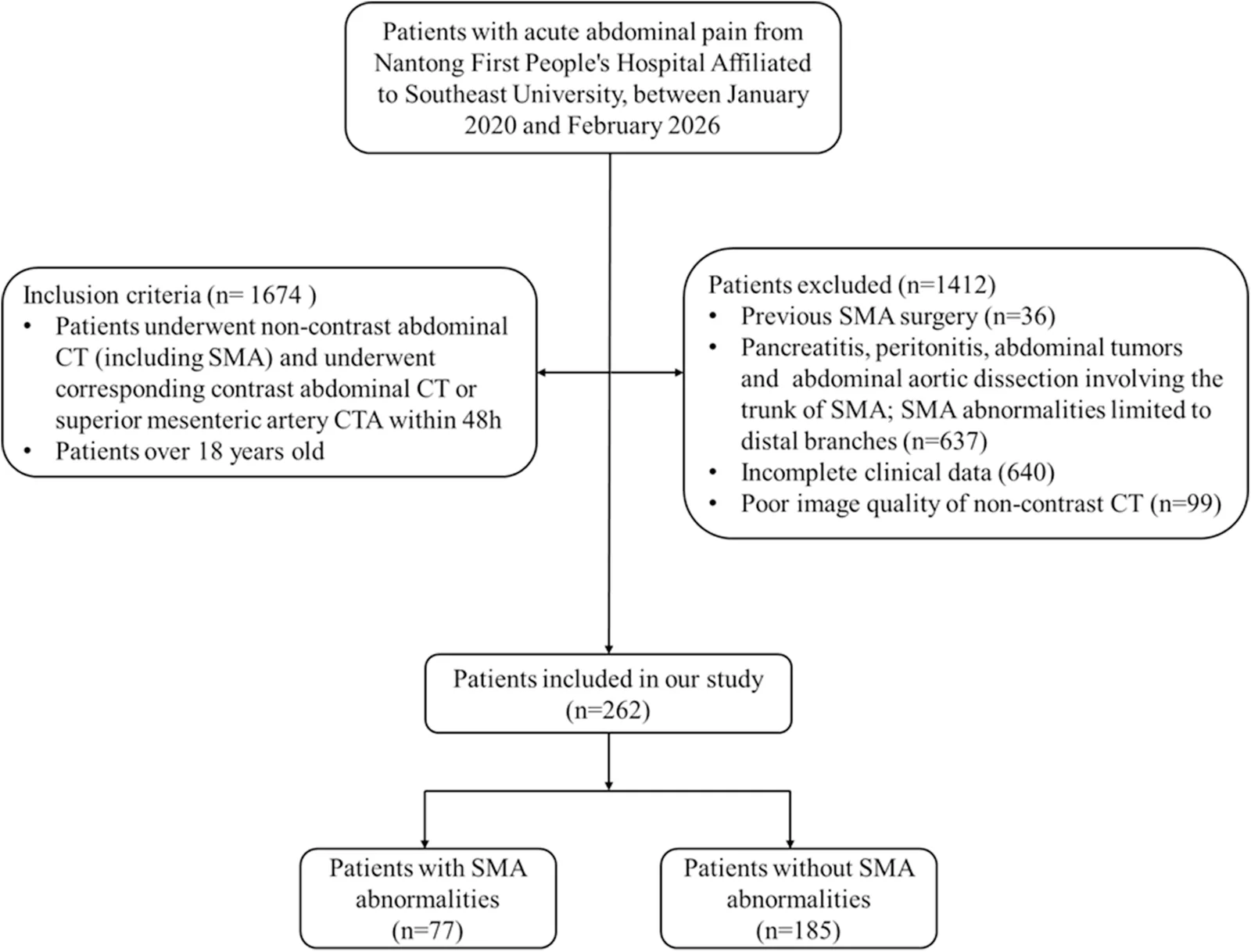
Flowchart of patient enrollment. CT, computed tomography; CTA, computed tomography angiography; SMA, superior mesenteric artery.

Among the 1,674 patients initially screened, 262 were ultimately enrolled, including 77 in the SMA abnormality group and 185 in the control group (Figure 2). The SMA abnormality group comprised 59 cases of isolated SMAD, of which 11 were dissecting aneurysms and 18 were SMAT. Subgroup analyses showed that FAI was significantly higher in both SISMAD and SMAT than in controls (median, −62.05 and −67.58 HU, respectively, vs. −85.05 HU; both *P* < 0.001). Although FAI was numerically lower in SMAT than in SISMAD, the between-subgroup difference was not statistically significant (median, −67.58 vs. −62.05 HU; *P* = 0.150). Compared to the control group, the SMA abnormality group had a significantly higher proportion of male patients and a greater prevalence of smoking history. In addition, laboratory measurements showed elevated levels of several biomarkers in the SMA abnormality group, including WBC count and hsCRP, D-dimer, and lipoprotein(a) levels. These differences were statistically significant (all *P* < 0.05), as shown in Table 2. The final diagnoses among the 185 controls were gallstones, cholecystitis, or intrahepatic cholangitis (*n* = 42); liver abscess (*n* = 2); intestinal obstruction (*n* = 4); peptic ulcer (*n* = 25); gastritis or colitis (*n* = 68); colorectal cancer (*n* = 11); appendicitis (*n* = 2); renal cell carcinoma, angiomyolipoma, or renal calculi (*n* = 9); renal infarction or severe renal artery stenosis (*n* = 2); pancreatic cancer (*n* = 3); and nonspecific abdominal pain (*n* = 17).

**Table 2:** Baseline characteristics of the study population.

Characteristic	Total (*n* = 262)	SMA Abnormalities group (*n* = 77)	Control group (*n* = 185)	*P* Value
Age (years)	61 (53, 74)	65 (54, 72)	61 (53, 74)	0.584
Gender				0.001
Female	75 (28.6%)	11 (14.3%)	64 (34.6%)	
Male	187 (71.4%)	66 (85.7%)	121 (65.4%)	
BMI (kg/m^2^)				0.873
<18.5	9 (3.4%)	3 (3.9%)	6 (3.2%)	
18.5–23.9	130 (49.6%)	37 (48.1%)	93 (50.3%)	
24.0–27.9	98 (37.4%)	31 (40.3%)	67 (36.2%)	
≥28.0 (China)	25 (9.5%)	6 (7.8%)	19 (10.3%)	
Hypertension				0.059
No	136 (51.9%)	33 (42.9%)	103 (55.7%)	
Yes	126 (48.1%)	44 (57.1%)	82 (44.3%)	
Diabetes				0.698
No	214 (81.7%)	64 (83.1%)	150 (81.1%)	
Yes	48 (18.3%)	13 (16.9%)	35 (18.9%)	
Cancer				0.196
No	204 (77.9%)	56 (72.7%)	148 (80.0%)	
Yes	58 (22.1%)	21 (27.3%)	37 (20.0%)	
Smoking history				<0.001
No	215 (82.1%)	52 (67.5%)	163 (88.1%)	
Yes	47 (17.9%)	25 (32.5%)	22 (11.9%)	
WBC (×10^9^/L)	6.1 (4.8, 7.5)	6.7 (5.1, 8.2)	5.9 (4.7, 7.0)	0.025
HsCRP (mg/L)	2.61 (0.20, 14.83)	9.72 (3.05, 33.37)	0.95 (0.10, 9.30)	<0.001
D-dimer (*μ*g/mL)	350 (210, 772.50)	420 (235, 980)	350 (202, 709)	0.048
Triglycerides (mmol/L)	1.31 (0.93, 1.84)	1.30 (0.80, 1.84)	1.31 (0.98, 1.83)	0.380
Total cholesterol (mmol/L)	4.31 ± 1.09	4.30 ± 1.08	4.31 ± 1.09	0.939
LDL (mmol/L)	2.67 ± 0.83	2.63 ± 0.86	2.68 ± 0.81	0.637
HDL (mmol/L)	1.15 (0.97, 1.40)	1.15 (0.97, 1.33)	1.16 (0.97, 1.40)	0.558
Apolipoprotein A-I (g/L)	1.11 (0.92, 1.30)	1.10 (0.89, 1.25)	1.11 (0.93, 1.31)	0.357
Apolipoprotein B (g/L)	0.82 (0.66, 1.00)	0.83 (0.69, 1.00)	0.80 (0.65, 1.00)	0.887
Lipoprotein a (mg/L)	130.5 (64.50, 263.25)	143 (96, 299)	123 (54, 257)	0.024

SMA, superior mesenteric artery; BMI, body mass index; WBC, white blood cell; hsCRP, high-sensitivity C-reactive protein; LDL, low-density lipoprotein; HDL, high-density lipoprotein.

### 3.2 Comparison of conventional non-contrast CT parameters and the FAI between the two groups

The interobserver agreement for all CT parameters demonstrated excellent consistency. The *kappa* value for SMA wall calcification was 0.862 (95% confidence interval [*CI*], 0.768–0.956). The *ICC* for SMA-d was 0.973 (95% *CI*, 0.966–0.979), for SMA-mean was 0.932 (95% *CI*, 0.914–0.946), for AM-a was 0.987 (95% *CI*, 0.983–0.990), for the aortic diameter at the SMA origin was 0.944 (95% *CI*, 0.929–0.956), and for FAI was 0.983 (95% *CI*, 0.979–0.987). All *kappa* and *ICC* values were >0.8, indicating strong interobserver agreement in the assessment of conventional CT parameters and FAI, and demonstrating good measurement reproducibility.

To assess potential measurement bias related to scanner platform, we compared FAI values obtained with the Definition Flash and SOMATOM Force scanners across the entire cohort. FAI did not differ significantly between scanner platforms (median, −79.10 vs. −78.83 HU; *P* = 0.984).

Univariate analysis showed that the SMA abnormality group had significantly higher values for several parameters than the control group. Specifically, the SMA-d and aortic diameter at the SMA origin were significantly larger, and FAI values were significantly higher in the SMA abnormality group than in the control group. In addition, the prevalence of SMA wall calcification was significantly higher in the SMA abnormality group. All differences were statistically significant (all *P* < 0.05; Table 3).

**Table 3:** Comparison of conventional non-contrast CT parameters and FAI between the SMA abnormality group and control group.

Characteristic	Total (*n* = 262)	SMA abnormalities group (*n* = 77)	Control group (*n* = 185)	*P* Value
SMA-d (mm)	7.88 (6.75, 9.66)	10.25 (9.35, 11.35)	7.25 (6.50, 8.05)	<0.001
SMA-mean (HU)	44.06 ± 6.97	44.38 ± 7.35	43.93 ± 6.82	0.636
AM-a (°)	54.29 ± 22.08	57.44 ± 19.86	52.98 ± 22.87	0.137
Aortic diameter at SMA origin (mm)	21.63 ± 2.54	22.46 ± 2.26	21.28 ± 2.57	0.001
SMA wall calcification				<0.001
No	225 (85.9%)	52 (67.5%)	173 (93.5%)	
Yes	37 (14.1%)	25 (32.5%)	12 (6.5%)	
FAI (HU)	−78.98 (−89.58, −66.23)	−63.05 (−70.05, −48.40)	−85.05 (−93.25, −75.65)	<0.001

SMA, superior mesenteric artery; SMA-d, superior mesenteric artery diameter; SMA-mean: CT value of the superior mesenteric artery; AM-a: angle between the superior mesenteric artery and the abdominal aorta; FAI, fat attenuation index.

### 3.3 Multivariate logistic regression analysis and model construction

A multicollinearity analysis was conducted for the variables that demonstrated statistical significance with a *P* value < 0.1 in the univariate analysis. Each variable displayed a VIF of less than 5, which was lower than the established threshold of 10, suggesting the absence of significant multicollinearity (Table 4).

**Table 4:** Multicollinearity analysis of 11 variables after univariate analysis.

Variables	Tolerance	*VIF*
Gender	0.695	1.439
Hypertension	0.854	1.171
Smoking history	0.890	1.124
WBC	0.830	1.205
hsCRP	0.721	1.387
D-dimer	0.789	1.267
Lipoprotein a	0.971	1.029
SMA wall Calcification	0.767	1.304
SMA-d	0.532	1.879
Aortic diameter at the origin of SMA	0.727	1.376
FAI	0.725	1.379

WBC, white blood cell; hsCRP, high-sensitivity C-reactive protein; SMA, superior mesenteric artery; SMA-d, superior mesenteric artery diameter; FAI, fat attenuation index; VIF, variance inflation factor.

Binary multivariate logistic regression analysis was performed to identify independent predictors of SMA abnormalities. Four variables were identified as significant independent predictors: smoking history [odds ratio (OR) = 10.452; 95% *CI*, 2.528–43.219; *P* = 0.001], elevated hsCRP (OR = 1.012; 95% *CI*, 1.001–1.023; *P* = 0.033), increased SMA-d (OR = 4.414; 95% *CI*, 2.758–7.062; *P* < 0.001), and high FAI (OR = 1.092; 95% *CI*, 1.053–1.132; *P* < 0.001; Table 5). Although SMA wall calcification and aortic diameter at the SMA origin were significant in univariable analyses, neither was retained in the final model. During backward selection, both variables were removed after losing statistical significance (*P* > 0.05) following adjustment for the other covariates. Given that all *VIF* values were <2, their exclusion was unlikely to reflect multicollinearity and instead suggested limited independent predictive contribution. The findings from the logistic regression analysis led to the development of four distinct predictive models: ① Model A (baseline), smoking + hsCRP; ② Model B: smoking + hsCRP + SMA-d; ③ Model C: smoking + hsCRP + FAI; ④ Model D: smoking + hsCRP + SMA-d + FAI.

**Table 5:** Multivariate logistic regression analysis of independent predictors for SMA abnormalities.

Variables	B	SE	Wald	*P* value	OR	95% *CI*
Smoking history	2.347	0.724	10.500	0.001	10.452	2.528–43.219
hsCRP	0.012	0.006	4.564	0.033	1.012	1.001–1.023
SMA-d	1.485	0.240	38.323	<0.001	4.414	2.758–7.062
FAI	0.088	0.019	22.193	<0.001	1.092	1.053–1.132

hsCRP, high-sensitivity C-reactive protein; SMA-d, superior mesenteric artery diameter; FAI, fat attenuation index; OR, odds ratio; CI, confidence interval.

### 3.4 Incremental predictive value of the FAI

**Figure 3:**
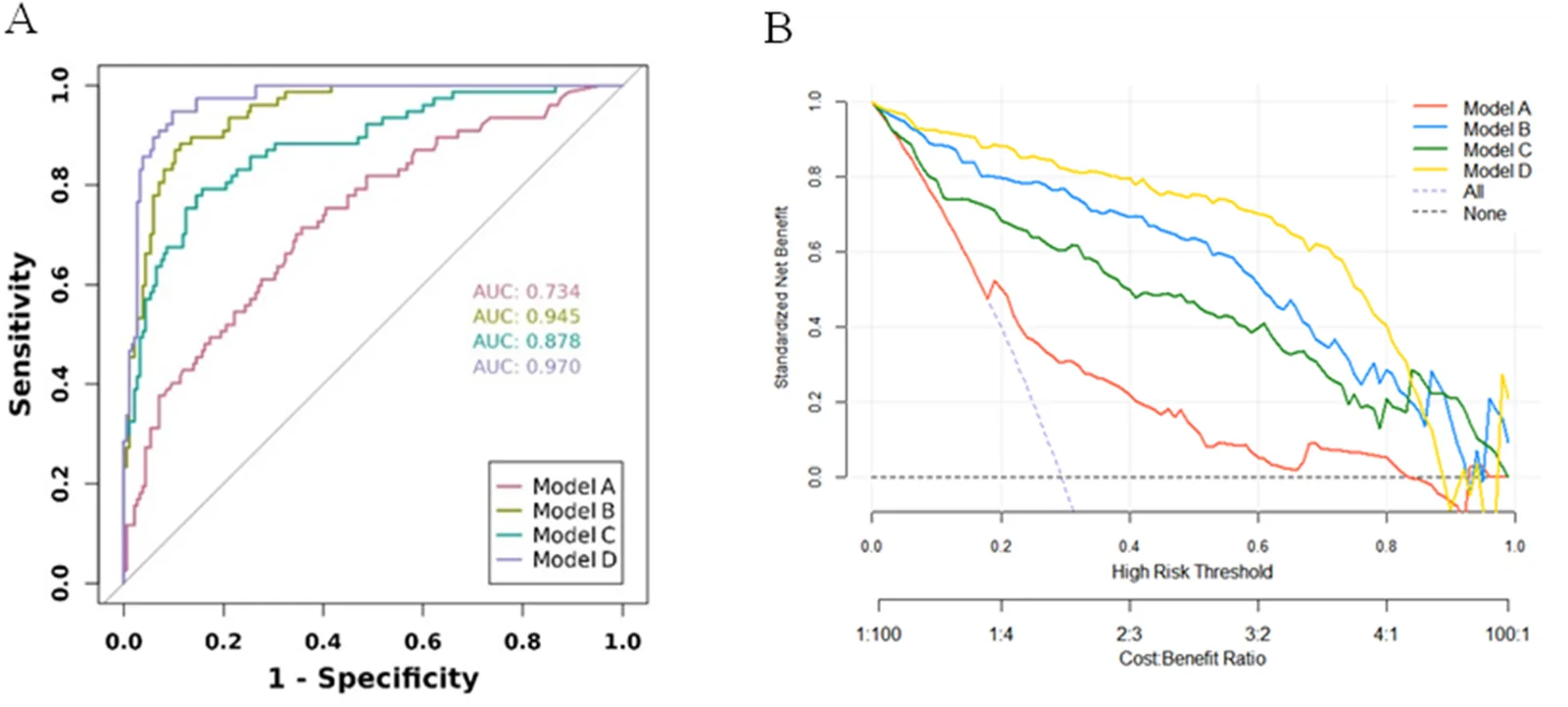
**(A)** Receiver operating characteristic (ROC) curve analysis of the predictive performance of four models for superior mesenteric artery abnormalities. The area under the ROC curve (AUC) for Model D was greater than that of the other models. **(B)** Decision curve analyses of the four models. Model D demonstrated the highest overall net benefit across a threshold probability range of 0%–91%.

ROC curve analysis demonstrated that Model D achieved superior predictive performance compared to Models A, B, and C (Table 6, Figure 3A). The addition of the FAI to Model A (Model C) increased the AUC from 0.734 to 0.878. Similarly, adding the FAI to Model B (Model D) increased the AUC from 0.945 to 0.970. Compared with baseline Models A and C, Model B, which incorporated SMA-d, also showed significantly improved discrimination (AUC = 0.945). At the optimal cutoff value of 0.2174, Model D achieved a sensitivity of 94.81% and a specificity of 90.27%. In addition, Model D demonstrated strong performance in correctly classifying both positive and negative cases. The differences in AUCs among the four models were statistically significant (all *P* < 0.001).

**Table 6:** Predictive performance of different models for SMA abnormalities.

Model	AUC (95% CI)	*P*	Cutoff	SEN (%)	SPEC (%)	PPV (%)	NPV (%)
Model A	0.734 (0.667–0.801)	<0.001	0.2104	71.43	64.32	45.45	84.40
Model B	0.945 (0.919–0.971)	<0.001	0.3002	88.31	88.65	76.40	94.80
Model C	0.878 (0.831–0.925)	<0.001	0.3070	79.22	84.32	67.78	90.70
Model D	0.970 (0.952–0.988)	<0.001	0.2174	94.81	90.27	80.22	97.66

Model A: baseline model (Smoking + hsCRP); Model B: baseline model + SMA-d; Model C: baseline model + FAI; Model D: baseline model + SMA-d + FAI.

AUC, area under the curve; CI, confidence interval; SEN, sensitivity; SPEC, specificity; PPV, positive predictive value; NPV, negative predictive value.

The NRI and IDI were used to evaluate the reclassification performance (Table 7). Compared with Model A, Model D showed the highest NRI (0.780; 95% *CI*, 0.629–0.932; *P* < 0.001) and IDI (0.573; 95% *CI*, 0.497–0.649; *P* < 0.001), indicating the greatest improvement in reclassification and discrimination. Models B and C also demonstrated significant improvements compared to Model A. However, the incremental predictive value of Model C over Model A (NRI = 0.457; IDI = 0.295; both *P* < 0.001) was smaller than that of Model B over Model A (NRI = 0.657; IDI = 0.460; both *P* < 0.001). Compared to Model B, Model D demonstrated a modest but statistically significant improvement in both NRI (0.220; 95% *CI*, 0.093–0.346; *P* < 0.001) and IDI (0.113; 95% *CI*, 0.069–0.157; *P* < 0.001). In contrast, a comparison between Models C and B showed no significant difference in the NRI (0.169; 95% *CI*, 0.016–0.354; *P* = 0.073), although the IDI was significantly higher in Model C (0.165; 95% *CI*, 0.071–0.259; *P* < 0.001).

**Table 7:** Incremental value of FAI based on reclassification and discrimination (NRI and IDI).

Model	Comparison	NRI (95%CI)	*P* Value	IDI (95%CI)	*P* Value
Model A		–			
Model B	VS. Model A	0.657 (0.492–0.822)	<0.001	0.460 (0.383–0.537)	<0.001
Model C	VS. Model A	0.457 (0.302–0.611)	<0.001	0.295 (0.228–0.362)	<0.001
	VS. Model B	0.169 (0.016–0.354)	0.073	0.165 (0.071–0.259)	<0.001
Model D	VS. Model A	0.780 (0.629–0.932)	<0.001	0.573 (0.497–0.649)	<0.001
	VS. Model B	0.220 (0.093–0.346)	<0.001	0.113 (0.069–0.157)	<0.001
	VS. Model C	0.427 (0.275–0.580)	<0.001	0.278 (0.211–0.346)	<0.001

Model A: Baseline model (Smoking + hsCRP); Model B: Baseline model + SMA-d; Model C: Baseline model + FAI; Model D: Baseline model + SMA-d + FAI.

NRI, net reclassification index; IDI, integrated discrimination index.

### 3.5 Clinical utility

**Figure 4:**
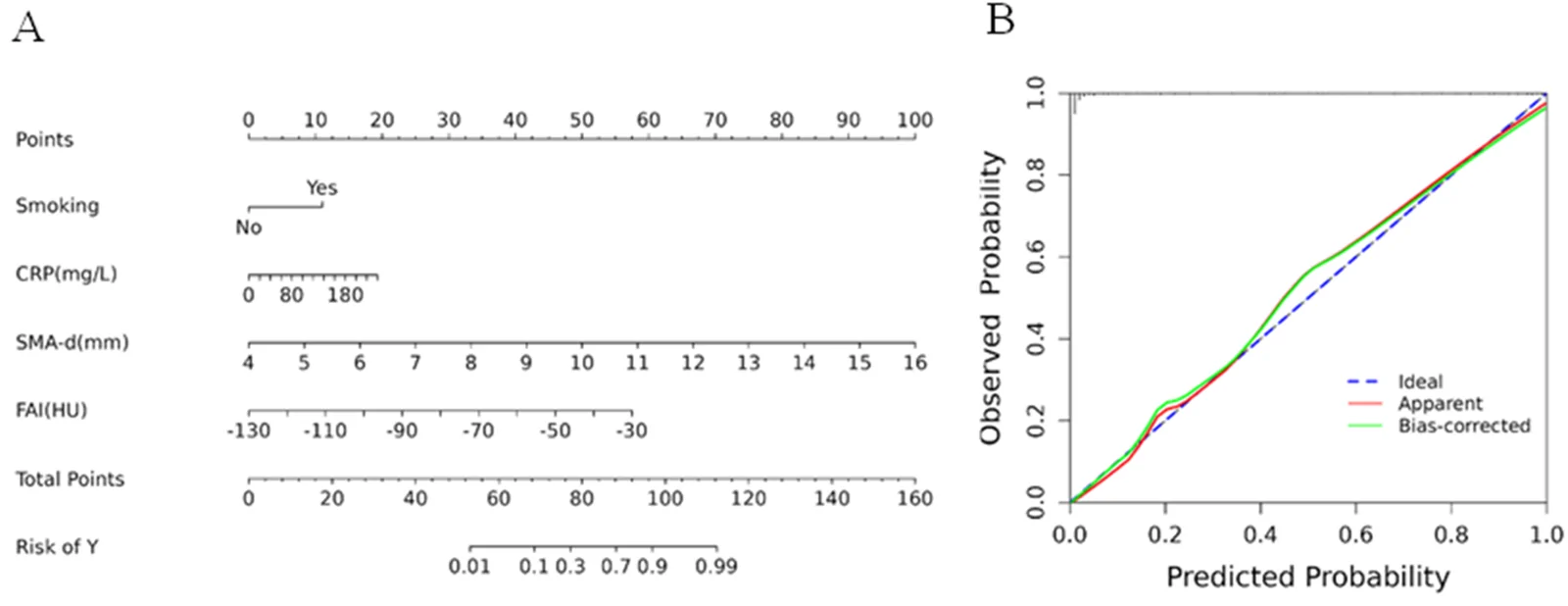
**(A)** nomogram for predicting the risk of superior mesenteric artery (SMA) abnormalities, visualizing the established prediction model incorporating independent risk factors. **(B)** Calibration curve of the nomogram. The ideal curve (dashed line), calibration curve (green solid line), and prediction curve (red solid line) are closely aligned, indicating good predictive performance of the model. CRP, C-reactive protein; FAI, fat attenuation index; SMA-d, superior mesenteric artery diameter.

**Figure 5:**
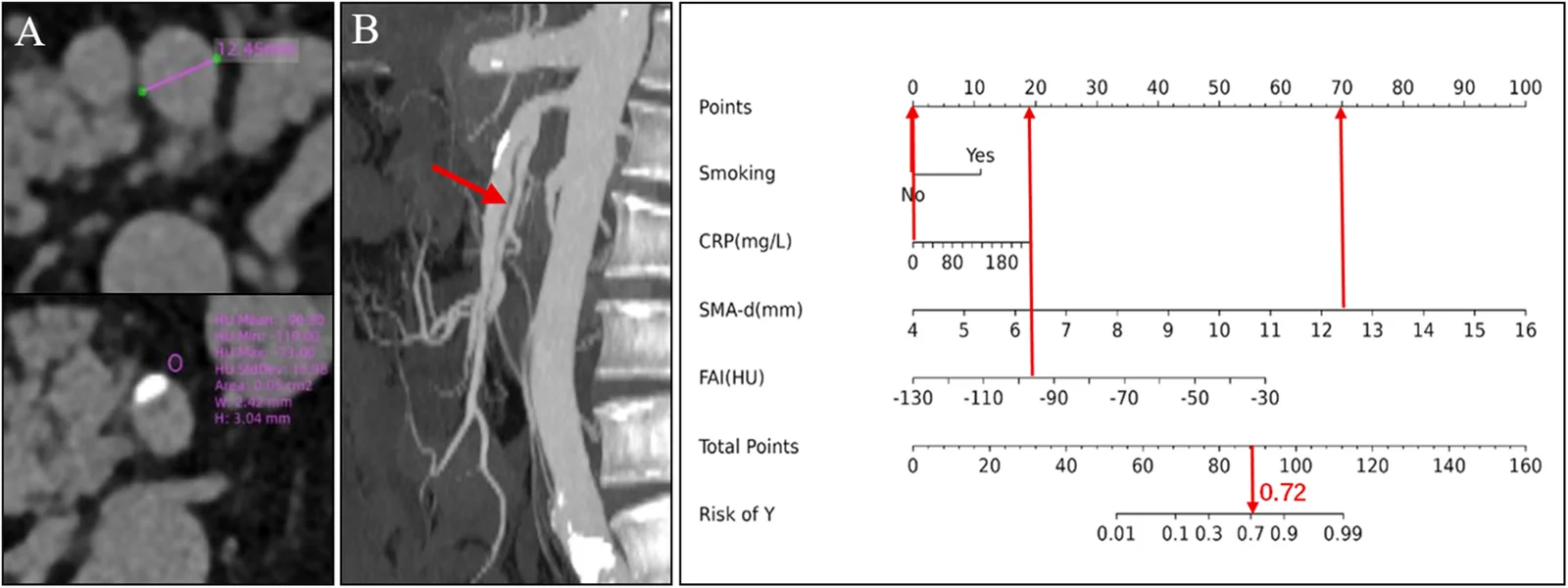
Illustration of the process for calculating the risk of superior mesenteric artery (SMA) abnormalities using the nomogram. A 53-year-old male patient was admitted with acute abdominal pain and no smoking history. Their hsCRP level on admission was 0.73 mg/L. **(A)** On the non-contrast abdominal computed tomography image, SMA-d was measured as 12.45 mm, and the mean FAI was −95.93 HU. The corresponding total nomogram score was 89, with a predicted risk of SMA abnormality of approximately 0.72. **(B)** The sagittal maximum intensity projection image of the SMA on computed tomography angiography demonstrated SMA dissection (red arrow).

The clinical utility of the models was evaluated by DCA (Figure 3B). The results showed that Model D, which included smoking history, hsCRP level, SMA-d, and the FAI, provided the highest net clinical benefit across a threshold probability range of 0%–91%. A nomogram was constructed based on Model D (Figure 4A). This nomogram allows clinicians to calculate total scores according to the weighted contribution of each predictor rather than relying on any single variable. The sum of these scores corresponds to the estimated risk of SMA abnormalities on the risk axis of the nomogram. A predicted probability at or above the optimal threshold of 0.2174 was used to prompt further vascular imaging with CTA or CECT. The nomogram calibration curve displayed a strong alignment with the reference line (Figure 4B). The results of the Hosmer–Lemeshow test corroborated this finding, revealing no statistically significant difference between the predicted probabilities generated by the model and the actual observed outcomes (*χ*^2^ = 4.756; *P* = 0.783). After optimism correction, the AUC was 0.966 (95% *CI*, 0.949–0.986), the calibration slope was 0.978, and the Brier score was 0.060 (95% *CI*, 0.036–0.079). An example of the nomogram applied to clinical practice is shown in Figure 5.

## 4 Discussion

This study evaluated the predictive value of the perivascular FAI around the SMA, derived from non-contrast CT, for identifying SMA abnormalities in patients with acute abdominal pain and developed a nomogram incorporating smoking history, hsCRP, SMA-d, and FAI. The discussion focuses on three principal findings.

### 4.1 Clinical and conventional non-contrast CT features of SMA abnormalities

Smoking history and elevated hsCRP emerged as independent predictors of SMA abnormalities, consistent with previous evidence. Smoking is among the most common risk factors for isolated SMAD (23) and is strongly associated with elevated levels of inflammatory indicators, including hsCRP, interleukin-6, and glycoprotein acetylation as well as thrombotic markers such as fibrinogen (24–27). Yao et al. (28) reported a dose–response relationship between smoking and subclinical cardiovascular damage including inflammation, thrombosis, and asymptomatic atherosclerosis. This suggests that even a small increase in tobacco exposure may result in significant changes in the expression of these pathological markers. As a systemic marker of inflammation, hsCRP may promote atherogenesis by facilitating monocyte adhesion and migration within the vasculature (29) and inducing M1 macrophage polarization (30). Elevated hsCRP is also associated with cardiovascular disease risk (31, 32). However, direct evidence linking smoking, elevated hsCRP, and SMA atherosclerosis remains lacking, and further studies are needed to clarify the interrelationships among these factors.

Among non-contrast CT features, SMA-d directly quantifies vascular morphologic changes. False-lumen expansion in SMAD, aneurysmal dilatation, and compensatory proximal luminal enlargement associated with thromboembolism may all increase SMA-d. Previous studies have proposed reference indicators for the early detection of SMA abnormalities on non-contrast CT. Among these indicators, an increase in SMA-d and the presence of PFS have been highlighted as significant markers (14, 33). Lei et al. (34) suggested that in patients with unexplained abdominal pain, particularly in men around 50 years of age with hypertension, isolated SMAD should be suspected when the SMA-d is enlarged or when the maximal diameter is located at the level of the left renal vein on non-contrast CT, prompting further CECT or CTA. It should be noted that SMA-d may be influenced by individual anatomical variability, and early localized SMAD or thromboembolic disease may not result in significant diameter changes. Therefore, combination with other imaging indicators is necessary for accurate differentiation.

### 4.2 Biological basis and clinical significance of the FAI

Vascular inflammation plays a crucial role in the pathophysiology of aortic dissection, leading to compromised structural integrity of the aortic wall and progression of dissection (35). Vascular inflammation extending into the adjacent PVAT, manifests as increased CT attenuation of the adipose tissue. As a metabolically active tissue, PVAT regulates vascular physiological functions through the paracrine secretion of adipokines, which play a role in cardiovascular diseases. FAI, a noninvasive imaging biomarker, has been introduced as a surrogate marker of vascular inflammation (8, 36) and is widely used in cardiovascular research. For example, Baradaran et al. (37) revealed a significantly elevated mean FAI in patients experiencing ischemic stroke compared with that in asymptomatic individuals (−66.2 ± 19.2 HU vs. −77.1 ± 20.4 HU, *P* = 0.009). This suggested that alterations in fat attenuation in PVAT may be associated with the pathophysiology of ischemic strokes. Kuneman et al. (38) further showed that pericoronary fat attenuation may help distinguish acute from stable coronary conditions, supporting its potential utility in cardiovascular risk assessment. Similarly, the cardiovascular prevention literature identifies epicardial adipose tissue and hepatic steatosis as potential imaging markers of cardiovascular risk (39), underscoring the capacity of imaging to reveal novel risk indicators and strengthen primary and secondary prevention. By contrast, our study extended perivascular fat imaging to an acute-care setting, using it to screen for SMA abnormalities rather than to estimate population-level risk.

Beyond extending this concept from preventive imaging to acute care, our study advanced SMA fat imaging from qualitative assessment toward quantitative characterization. A previous study has found that PFS on non-contrast CT may serve as a marker for isolated SMAD (14). However, their study included only patients with SISMAD, excluded those with SMA embolism who may also present with PFS, and did not perform a quantitative analysis of PFS. In the present study, FAI provided a quantitative measure of adipose tissue surrounding the SMA, offering a more precise evaluation than the binary assessment of PFS used previously. FAI independently predicted SMA abnormalities. Although its association was weaker than that of SMA-d, FAI may capture the inflammatory component of vessel-wall injury. Compared with traditional inflammatory markers such as hsCRP, FAI has spatial specificity, directly localizing perivascular inflammation of the SMA and detecting early vascular wall instability, thereby providing an early warning signal for SMA abnormalities. Nevertheless, FAI alone did not reliably distinguish SISMAD from SMAT, possibly reflecting partially overlapping pathophysiologic mechanisms.

### 4.3 Predictive value of the FAI and clinical utility of the nomogram for SMA abnormalities

Model D, which incorporated all four predictors, showed the strongest overall performance for identifying SMA abnormalities in patients with acute abdominal pain, with excellent discrimination (AUC = 0.970) and calibration. Decision curve analysis indicated the greatest net benefit across threshold probabilities of 0% to 91%, suggesting potential utility across a broad range of clinical risk tolerances. Its performance approached that of the YOLOv8 deep-learning model developed by Mei et al. (2) for detecting SMA abnormalities on non-contrast CT (AUC = 0.990) and exceeded that of the clinical-radiomics model developed by Qiu et al. (40) for diagnosing mesenteric arterial embolism (training AUC = 0.94; validation AUC = 0.93). These cross-study comparisons should be interpreted cautiously because of differences in study populations and outcomes. Overall, FAI appears to be a complementary inflammatory imaging biomarker rather than a stand-alone screening test. Combining FAI with SMA-d and clinical variables may provide more informative risk assessment than any component alone.

The Model D nomogram translates the four predictors into a total-point score that can inform clinical decision-making. A predicted probability above the optimal cutoff of 0.2174 may prompt confirmatory vascular imaging with CTA or CECT. This threshold estimates only the likelihood of SMA abnormalities and does not exclude other causes of acute abdominal pain; evaluation for alternative diagnoses remains at the treating physician's discretion. Thus, the nomogram is intended to complement, not replace, clinical judgment.

Integrating the FAI into SMA risk prediction frameworks may facilitate personalized management of patients with acute abdominal pain. Future guidelines should consider incorporating the FAI as a biomarker to inform individualized diagnostic strategies. However, further prospective studies are required to determine whether FAI-guided diagnostic approaches can improve clinical outcomes. Moreover, with the expanding application of artificial intelligence and machine learning in cardiovascular disease assessment, future predictive models that incorporate FAI parameters may enable more accurate cardiovascular risk stratification and enhance the precision of clinical decision-making.

### 4.4 Limitations

This study has several limitations. First, one significant constraint is its execution at a single center, which inherently restricts the generalizability of the findings. The use of a relatively small sample size further compounded this issue as it may have led to potential selection bias. Such a bias can influence the results and limit the applicability of the conclusions drawn from this study, emphasizing the need for caution when interpreting the outcomes in a broader context. To establish the generalizability of the FAI as an indicator of SMA abnormalities in patients with acute abdominal pain, external validation and large-scale, multicenter, prospective studies are required. Second, FAI was averaged along the entire SMA trunk. Although inflammation is likely to be concentrated near the entry tear, false lumen, or lesion site, the model was designed for use on unenhanced CT before a definitive diagnosis was available; lesion-specific sampling would therefore have risked data leakage. Averaging measurements over the entire vessel may dilute focal inflammatory signals and bias the observed association toward the null, thereby underestimating the discriminative performance of FAI. In addition, the manual measurement of FAI is time-consuming, and future studies should adopt semi-automated or fully automated segmentation methods to extract PVAT parameters. Third, this study did not evaluate the predictive performance of the FAI for different subtypes of SMA abnormalities, which will be explored in future research.

## 5 Conclusions

Smoking history, elevated hsCRP levels, increased SMA-d, and high FAI were identified as independent predictors of SMA abnormalities in patients with acute abdominal pain. Although the FAI significantly improved the predictive performance of the baseline models, its incremental value was lower than that of SMA-d. A nomogram integrating these four indicators may optimize the diagnostic workflow for acute abdominal pain on non-contrast CT and provide decision-making support for timely intervention.

## Data Availability

The original contributions presented in the study are included in the article/Supplementary Material, further inquiries can be directed to the corresponding author.
